# Epigenetics in Atrial Fibrillation: Molecular Mechanisms and Therapeutic Avenues

**DOI:** 10.31083/RCM49551

**Published:** 2026-07-16

**Authors:** Xinyi Liu, Chaojun Yang, Yifan Huang, Bingxun Li, Jian Yang, Zhixing Fan

**Affiliations:** ^1^Department of Cardiology, the First College of Clinical Medical Sciences, China Three Gorges University, 443003 Yichang, Hubei, China; ^2^Hubei Key Laboratory of Ischemic Cardiovascular Disease, 443003 Yichang, Hubei, China; ^3^Hubei Provincial Clinical Research Center for Ischemic Cardiovascular Disease, 443003 Yichang, Hubei, China

**Keywords:** atrial fibrillation, epigenetics, DNA methylation, histone modification, RNA small untranslated, atrial remodeling, molecular targeted therapy

## Abstract

Atrial fibrillation (AF) represents the most common sustained cardiac arrhythmia in clinical practice and imposes a growing global disease burden. However, current therapeutic strategies remain considerably limited in efficacy, durability, and safety. Recently, the role of epigenetic mechanisms in the pathogenesis of AF, as key regulators of atrial structural and electrical remodeling, has garnered considerable attention. This review systematically elucidates the central role of multiple epigenetic regulatory mechanisms, including deoxyribonucleic acid (DNA) methylation, histone modifications, non-coding ribonucleic acids (RNAs) (microRNAs, long non-coding RNAs, circular RNAs, and transfer RNA-derived small RNAs), chromatin remodeling, and RNA methylation, in the atrial structural and electrical remodeling underlying AF. These reversible molecular modifications are widely implicated in the pathophysiological processes of AF through the regulation of key gene expression. In addition, circulating epigenetic markers (e.g., methylated DNA fragments and specific miRNAs) show substantial potential as biomarkers for AF diagnosis, risk stratification, and relapse prediction. Furthermore, targeted epigenetic intervention strategies (*e*.*g*., histone deacetylase (HDAC) inhibitors, demethylating agents) offer novel avenues for the precision therapy of AF. Thus, this review seeks to comprehensively summarize recent advances in the epigenetic regulation of AF pathogenesis, provide a theoretical basis for an in-depth understanding of the underlying mechanisms, and inform the development of innovative preventive and therapeutic strategies.

## 1. Introduction

Atrial fibrillation (AF), the most prevalent sustained cardiac arrhythmia, affects approximately 52.6 million people globally and markedly elevates the risk of stroke and cardiovascular mortality, with a rising burden particularly among women [1]. Fuelled by population aging and improved survival among patients with cardiovascular disease, the global burden of AF continues to rise [2]. However, therapeutic strategies for AF, including stroke-preventive anticoagulation, antiarrhythmic drugs and catheter ablation [3,4], are hampered by proarrhythmic effects and high post-ablation recurrence rates, highlighting the unmet clinical need for safer and more durable therapeutic alternatives.

The role of epigenetic mechanisms in AF pathogenesis has grown increasingly prominent in recent years. Epigenetic regulation encompasses processes such as DNA methylation, histone modifications, non-coding RNA activity, chromatin remodeling and RNA methylation [5,6,7]. It modulates critical pathophysiological pathways, including ion channel function, fibrotic remodeling and inflammatory responses, thereby driving the structural and electrical remodeling that initiates and sustains AF [5]. Circulating epigenetic biomarkers such as non-coding RNAs and methylated DNA in peripheral blood, show great promise for the diagnosis, risk stratification and recurrence prediction of AF [5]. Meanwhile, targeted interventions, including histone deacetylase inhibitors and miRNA-targeted therapeutics, have emerged as promising novel therapeutic strategies [8,9,10]. Therefore, an enhanced understanding of the epigenetic regulatory network in AF may facilitate early diagnosis, precise risk stratification and personalized therapeutic regimens, which in turn could improve the clinical outcomes of patients. Thus, elucidating the epigenetic mechanisms underlying AF is of considerable clinical importance for the prevention and treatment of the disease.

This review systematically elucidates epigenetic regulation in the atrial remodeling associated with AF, focusing on DNA methylation, histone modifications, non-coding RNAs, chromatin remodeling and RNA methylation. It also assesses their potential as diagnostic biomarkers and therapeutic targets, with the aim of providing insights to advance precision prevention and treatment of AF.

## 2. AF: Electrical and Structural Remodeling and Atrial Cardiomyopathy

AF is characterized by progressive cardiac remodeling, a process encompassing distinct yet interconnected structural and electrical alterations [11]. Structural remodeling refers to pathological changes that form a vulnerable anatomical substrate, which is histologically characterized by myocardial fibrosis, increased intercellular spacing, myofibrillar degeneration, and decreased nuclear density [12]. These changes correlate with alterations in atrial dimensions, cellular ultrastructure, and connexin expression profiles, ultimately leading to atrial dilation and, most critically, impaired electrical conduction [12]. In parallel, electrical remodeling involves ion channel dysfunction, reduced conduction velocity, and electrogram fractionation, fostering a proarrhythmic functional milieu that triggers and sustains the arrhythmia [11,12]. These two processes form a vicious cycle: structural damage induces electrical instability, which in turn exacerbates structural injury. Epigenetic mechanisms mediate both structural and electrical remodeling in AF by regulating genes critical to fibrotic, inflammatory, and electrophysiological pathways, thereby driving disease progression.

These structural and electrical alterations are now recognized as key components of a broader clinical pathological entity, atrial cardiomyopathy (AtCM) [13,14]. According to the latest consensus, AtCM is defined as a graded disorder encompassing structural, architectural, contractile, and electrophysiological changes affecting the atria [13]. Importantly, AtCM can exist independently of AF and serves as a substrate that predisposes to arrhythmia development, while AF itself accelerates AtCM progression, creating a self-perpetuating cycle [15]. Central to this cycle are the interconnected processes of inflammation, oxidative stress, and fibrosis, which not only drive structural and electrical remodeling but also underlie the prothrombotic alterations associated with the atrial substrate [15,16]. Advances in electrocardiography and multimodality imaging now enable non-invasive detection of AtCM, facilitating early risk stratification and clinical management [17].

## 3. DNA Methylation and AF

**Fig. 1.**
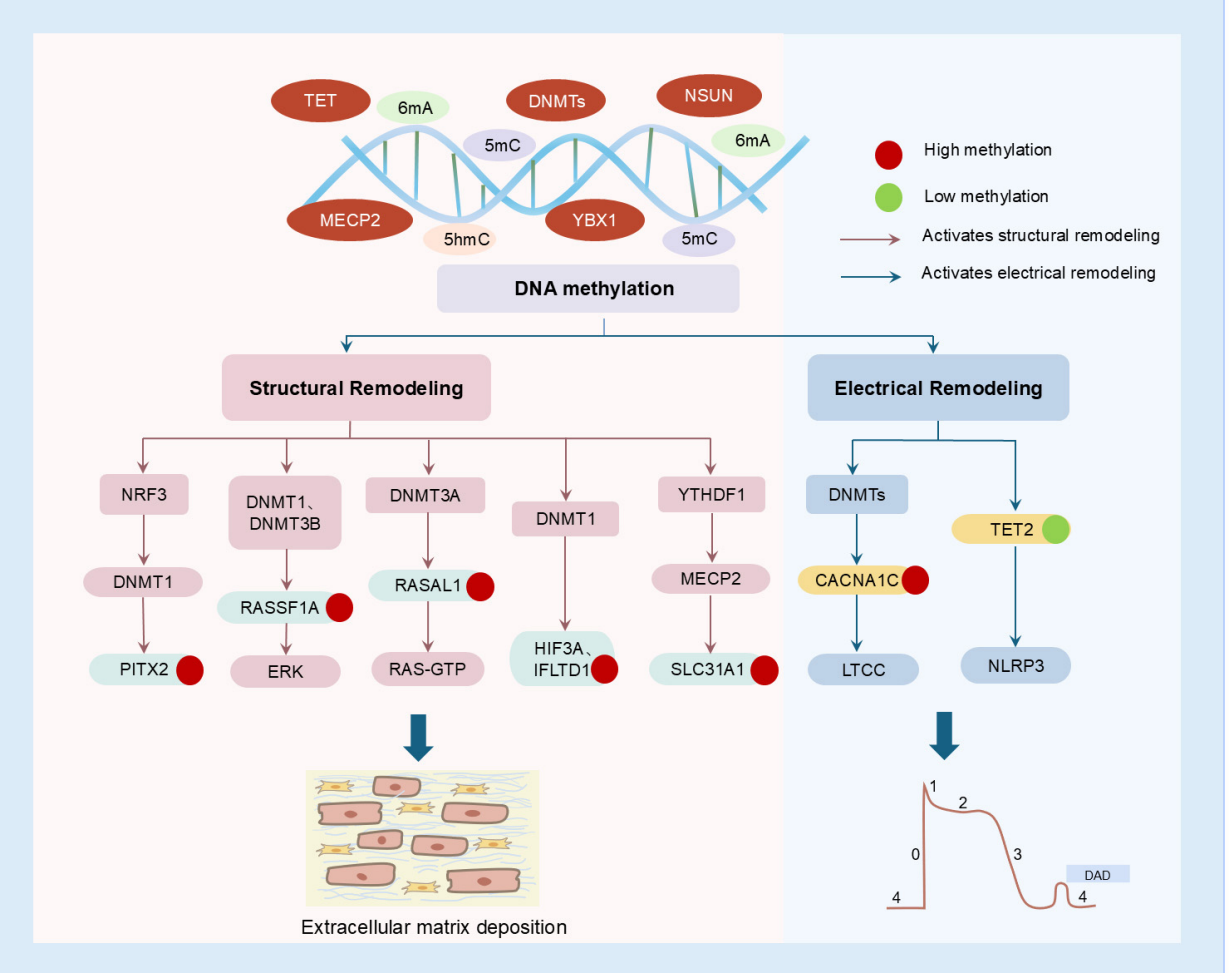
**Mechanistic map of DNA methylation regulating structural and electrical remodeling**. Abbreviations are defined as follows: 5mC, 5-methylcytosine; 6mA, N6-methyladenine; 5hmC, 5-hydroxymethylcytosine; *CACNA1C*, calcium voltage-gated channel subunit alpha1 C; DNMT1, DNA methyltransferase 1; DNMT3A, DNA methyltransferase 3 alpha; DNMT3B, DNA methyltransferase 3 beta; DNMTs, DNA methyltransferases; ERK, extracellular signal-regulated kinase; HIF2A, hypoxia inducible factor 2 alpha;* IFLTD1*, refer to LMNTD1 (Lamin Tail Domain Containing 1) in current gene nomenclature; LTCC, L-type calcium channel; *MECP2*, methyl-CpG binding protein 2; MYH7, myosin heavy chain 7; NLRP3, NLR family pyrin domain containing 3; NRF3, nuclear respiratory factor 3; NSUN2, NOP2/Sun RNA methyltransferase 2; RAS-GTP, RAS guanosine-5′-triphosphate; *RASSF1A*, Ras association domain family member 1;* SLC31A1*, solute carrier family 31 member 1; TET1, tet methylcytosine dioxygenase 1; YBX1, Y-box binding protein 1; *YTHDF1*, YTH N6-methyladenosine RNA binding protein F1; *PITX2*, paired like homeodomain 2; *RASAL1*, RAS protein activator like 1.

Methylation is the process by which a methyl group is added to the fifth carbon atom of cytosine, forming 5-methylcytosine (5mC). This process is catalyzed by DNA methyltransferases (DNMTs) and exerts a key role in the epigenetic regulation of gene expression [18]. Beyond 5mC, other DNA modifications such as 5-hydroxymethylcytosine (5hmC) and N^6^-methyladenine (6mA) mediate gene regulatory processes. Although research into these modifications in the cardiovascular field is far less extensive than that into 5mC, they also exert regulatory effects on gene expression [19,20]. Typically, promoter hypermethylation suppresses transcriptional activity, while hypomethylation enhances this process. Methylation status is dynamically controlled by “writers” (DNMT, NSUN, TRDMT), “erasers” (TET, ALKBH1) and “readers” (*MECP2*, ALYREF, YBX1) [21]. In AF, pathological stimuli such as chronic hypoxia have been reported to induce abnormal DNA methylation, which modulates the expression of genes implicated in multiple pathological processes, including extracellular matrix deposition, endothelial-to-mesenchymal transition, ion channel dysfunction and systemic inflammation, ultimately contributing to cardiac fibrosis and aberrant electrical activity (Fig. 1; Table 1, Ref. [22,23,24,25,26,27,28,29,30,31]).

**Table 1.** **DNA methylation changes associated with AF and their functional roles**.

Target gene/Region	Study model	Methylation status	Target gene/Pathways regulated	Net Effect on pathology (Promotes)	References
*PITX2* Promoter Region	Human left atrial tissue, Spontaneously hypertensive rat model	High methylation	Suppresses *PITX2* transcription	Structural Remodeling	[22,23]
*RASSF1A* Promoter Region	Chronic hypoxia mouse model, Cardiac fibroblasts	High methylation	Suppresses target gene expression and activates the Ras/ERK signaling pathway	Structural Remodeling	[24,25]
*RASAL1* Promoter Region	Human coronary artery endothelial cells, End-stage heart failure patients	High methylation	Suppresses target gene expression and activates the Ras-GTP signaling pathway	Structural Remodeling	[26]
*HIF3A*/*IFLTD1* Promoter Region	Mouse TAC model, DNMT1 heterozygous mutant mice	High methylation	Suppresses target gene expression and activates the HIF signaling pathway	Structural Remodeling	[28]
*SLC31A1* Promoter Region	Mouse CF model, Human atrial tissue	High methylation	Leads to mitochondrial copper depletion and glycolytic pathway reprogramming	Structural Remodeling	[27]
*CACNA1C* Intron 30 Region	AF patient tissue, Cell models	High methylation	Suppresses *CACNA1C* expression	Electrical Remodeling	[29]
*TET2*	*TET2*-deficient clonal homeostasis model	Low methylation	Activates NLRP3 inflammasome and CaMKII, disrupts calcium homeostasis	Electrical Remodeling	[30,31]

Abbreviations are defined as follows: AF, Atrial Fibrillation; *CACNA1C*, Calcium Voltage-Gated Channel Subunit Alpha1 C; CF, Cardiac Fibroblasts; ERK, Extracellular Signal-Regulated Kinase; GTP, Guanosine Triphosphate; HIF, Hypoxia Inducible Factor; *HIF3A*, hypoxia inducible factor 3 subunit alpha; *IFLTD1*, refer to LMNTD1 (Lamin Tail Domain Containing 1) in current gene nomenclature; *PITX2*, Paired Like Homeodomain 2; *RASAL1*, RAS Protein Activator Like 1; *RASSF1A*, Ras Association Domain Family Member 1; *SLC31A1*, Solute Carrier Family 31 Member 1; TAC, Transverse Aortic Constrictio; NLRP3, NLR family pyrin domain containing 3; DNMT1, DNA methyltransferase 1; *TET2*, tet methylcytosine dioxygenase 2; CaMKII, Calcium/calmodulin-dependent protein kinase II.

### 3.1 DNA Methylation Associated With AF Structural Remodeling

DNA methylation contributes to atrial structural remodeling in AF by regulating the expression of specific target genes. Left atrial tissue from AF patients exhibits reduced expression of *PITX2*, a key gene located in the 4q25 locus associated with increased AF risk. This downregulation is directly linked to transcriptional repression caused by hypermethylation of the *PITX2* promoter [22,23]. Evidence suggests that the transcription factor Nrf3 may promote fibrosis by recruiting DNA methyltransferase 1 (DNMT1) to the *PITX2* promoter region, thereby increasing local methylation levels and silencing gene expression [32]. Beyond *PITX2*, multi-omics studies have identified other genes associated with AF. For instance, hypermethylation at specific cytosine-guanine (CpG) sites in the titin (*TTN*) gene upregulates its expression and reduces AF risk [33], while aberrant activation of the DNA methyltransferase DNA methyltransferase 3 beta (DNMT3B) in valvular AF may exacerbate fibrosis via hypomethylation of profibrotic collagen genes [34].

Cardiac fibrosis, characterized by excessive extracellular matrix (ECM) deposition, is a core pathological feature of atrial structural remodeling [35], and DNA methylation contributes to this process through multiple signaling pathways. Chronic hypoxia can upregulate the expression of DNMT1 and DNMT3B in fibroblasts, leading to hypermethylation of the *RASSF1A* promoter region, subsequent activation of the extracellular signal-regulated kinase (ERK) pathway, and increased collagen I production [24]. DNMT3A also promotes fibroblast activation and proliferation by activating the Ras/extracellular signal-regulated kinase (ERK) pathway via methylation of *RASSF1A* [25]. Transforming growth factor-β1 (TGF-β1) induces methylation of the *RASAL1* gene promoter in endothelial cells and activates Ras-GTP signaling, contributing to endothelial-to-mesenchymal transition (EndMT) and fibrosis, a mechanism that has also been validated in patients with end-stage heart failure [26]. Recent studies have also revealed that YTH N6-methyladenosine RNA binding protein F1 (*YTHDF1*) promotes methyl-CpG binding protein 2 (*MeCP2*) translation, causing hypermethylation of the copper transporter gene solute carrier family 31 member 1 (*SLC31A1*), mitochondrial copper depletion, and enhanced glycolysis in fibroblasts, thereby driving cardiac fibrosis [27]. In pressure overload models, hypermethylation of *Hif3a* and *Ifltd1* is linked to atrial enlargement and fibrosis, a process that is attenuated in DNMT1 heterozygous mutant mice, implicating DNMT1 as a key regulator in this regulatory pathway [28].

### 3.2 DNA Methylation Associated With AF Electrical Remodeling

DNA methylation contributes to AF atrial electrical remodeling through both cardiac-intrinsic and systemic pathways. At the myocardial level, the calcium channel gene calcium voltage-gated channel subunit alpha1 C (*CACNA1C*) is regulated by methylation. In AF patients, specific CpG sites within intron 30 show significantly increased methylation, which may impair enhancer and transcriptional activity, leading to calcium channel dysfunction and the development of atrial electrical remodeling [29]. Systemically, *TET2*-deficient clonal hematopoiesis increases susceptibility to AF and promotes electrical instability by activating the NLRP3 inflammasome, which disrupts cardiomyocyte calcium handling via calcium–calmodulin (CaM)-dependent protein kinase II (CaMKII); this pathway can be inhibited by NLRP3 antagonists [30,31].

### 3.3 Potential Biomarkers and Therapeutic Targets for DNA Methylation in AF

Aberrant DNA methylation patterns in AF not only contribute to disease pathogenesis but also hold substantial translational potential as diagnostic and therapeutic tools. Several candidates illustrate this diagnostic potential: The pathogenic hypermethylation of *PITX2* and *CACNA1C* establishes them as direct therapeutic targets, with *PITX2* being a clinically relevant candidate due to its confirmed reversibility by decitabine. For non-invasive detection, methylation patterns in CDK5R1, GSE1, HSPG2, and WDFY3 from peripheral blood represent a promising approach [36]. Beyond diagnosis, the hypermethylation of MED1 and MED23 may serve as prognostic indicators for early-onset or progressive AF [37]. However, the current research in this field remains fragmented, as most studies only report correlative associations in individual cohorts. A critical future direction is to move beyond simple correlation by establishing the causal roles of these methylation events in AF persistence and progression via functional studies and multi-omics integration in well-phenotyped patient cohorts.

## 4. Histone Modification and AF

**Fig. 2.**
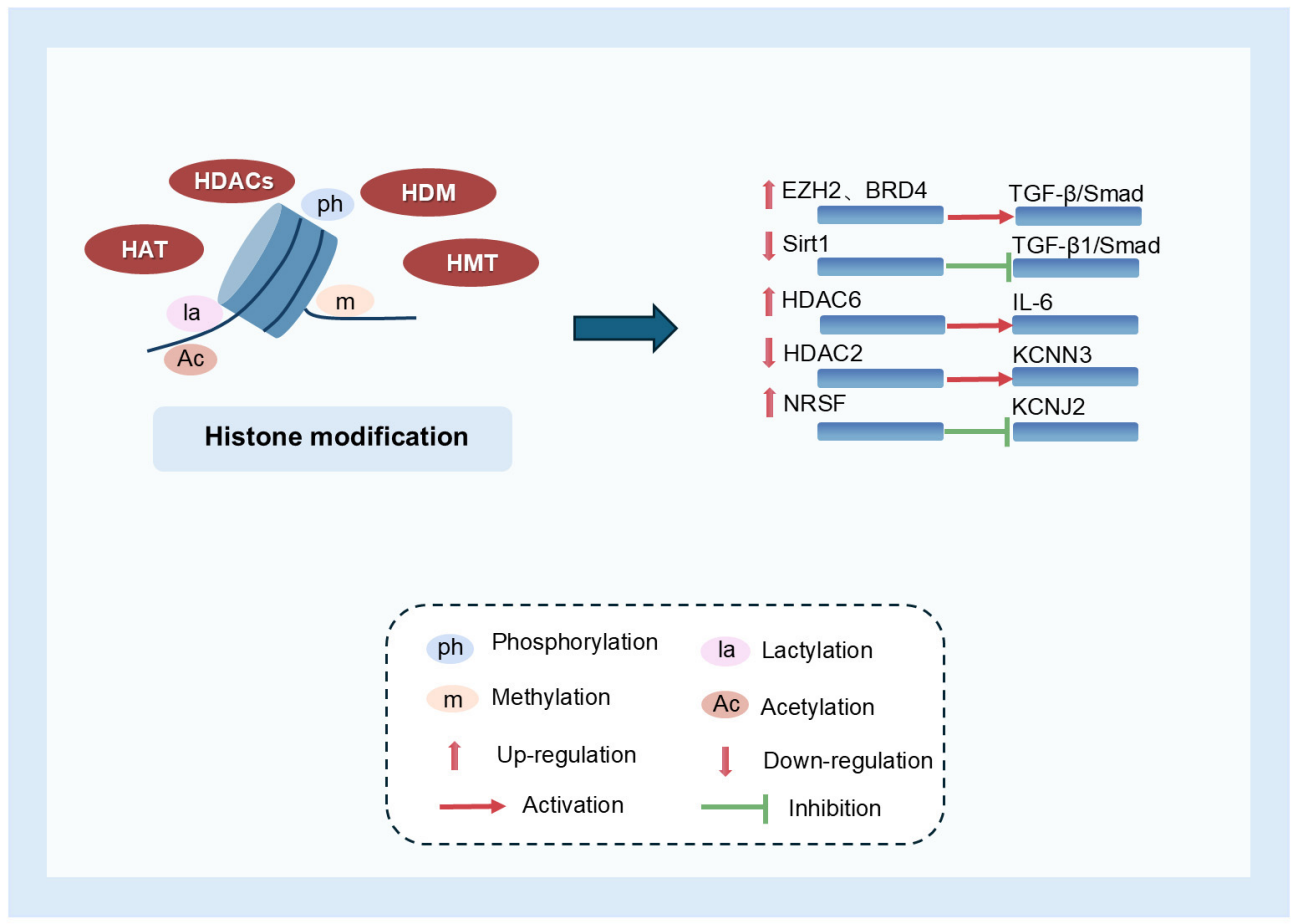
**A map of epigenetic regulatory mechanisms of histone modifications in the development of AF**. Abbreviations are defined as follows: *BRD4*, bromodomain containing 4; *EZH2*, enhancer of zeste 2 polycomb repressive complex 2 subunit; HATs, histone acetyltransferases;* HDAC2*, histone deacetylase 2; HDAC6, histone deacetylase 6; HDACs, histone deacetylases; HDM, histone demethylase; HMT, histone methyltransferase; IL-6, interleukin 6; *KCNJ2*, potassium inwardly rectifying channel subfamily J member 2; *KCNN3*, potassium calcium-activated channel subfamily N member 3; NRSF, neuron-restrictive silencer factor (also known as REST); *Sirt1*, sirtuin 1; TGF-β1, transforming growth factor beta 1.

Histone modification represents one of the core mechanisms underlying epigenetic regulation. It dynamically modulates chromatin architecture and gene transcriptional activity via chemical modifications on histone tails, including acetylation, methylation, phosphorylation, ubiquitination and lactylation [38,39]. These modifications are catalyzed by specific enzymes, including histone acetyltransferases (HATs), histone deacetylases (HDACs), histone methyltransferases (HMTs), and histone demethylases (HDMs), with their functional outcomes depend on the modified residues and modification states [40,41] (Fig. 2; Table 2, Ref. [42,43,44,45,46,47,48,49]). Histone modifications contribute to AF pathogenesis through multiple pathways. For instance, pathogenic TBX5 variants remodel the atrial transcriptome via histone mark alterations [50], while changes in chromatin accessibility at AF-associated gene loci are mediated by histone modifications such as H3K4me3 [51]. Thus, histone modifications drive AF pathogenesis by coordinately activating pro-fibrotic and pro-inflammatory pathways to promote structural remodeling, while dysregulating ion channel expression and calcium homeostasis to induce electrical instability.

**Table 2.** **Histone-Modifying enzymes associated with AF and their functions**.

Histone-modifying enzymes/complexes	Study model	Molecular changes in AF	Target gene/Pathways regulated	Net effect on pathology (Promotes)	References
*EZH2*	AF patient atrial tissue, Ang-II-induced mouse model	↑	Activates TGF-β/Smad signaling pathway	Structural Remodeling	[42]
*Sirt1*	AF patient atrial tissue	↓	Inhibits TGF-β/Smad signaling pathway	Structural Remodeling	[43]
*BRD4*	AF patient atrial tissue, TGF-β-induced fibrosis model	↑	Promotes fibrotic gene transcription; regulates EndMT	Structural Remodeling	[44,45]
HDAC4/HDAC5	AF patient atrial tissue	Phosphorylation and nuclear export	Relieves transcriptional repression of MEF2, inducing hypertrophy	Structural Remodeling	[47]
HDAC6	Chronic hypertensive transgenic mouse model	↑	Promotes IL-6 expression	Structural/Electrical Remodeling	[46]
HDAC2	AF patients with heart failure, rapid atrial pacing porcine model	↓	Promotes *KCNN3 *transcription	Structural/Electrical Remodeling	[49]
NRSF	AF patients and the porcine model	↑	Suppression of the *KCNJ2* gene expression	Electrical Remodeling	[48]

↑: upregulation; ↓: downregulation. Abbreviations are defined as follows: AF, Atrial Fibrillation; Ang-II, Angiotensin II; *BRD4*, Bromodomain Containing 4; EndMT, Endothelial-to-Mesenchymal Transition; *EZH2*, Enhancer of Zeste Homolog 2; HDAC, Histone Deacetylase; IL-6, Interleukin-6; *KCNJ2*, Potassium Inwardly Rectifying Channel Subfamily J Member 2; *KCNN3*, Potassium Calcium-Activated Channel Subfamily N Member 3; MEF2, Myocyte Enhancer Factor 2; NRSF, Neuron-Restrictive Silencer Factor; *Sirt1*, Sirtuin 1; TGF-β, Transforming Growth Factor Beta.

### 4.1 Histone Modifications Associated With AF Structure Remodeling

Histone modifications exert a critical role in AF-associated atrial structural remodeling, particularly in atrial fibrosis. Key regulators include the histone methyltransferase *EZH2*, which is upregulated in AF and induces fibroblast differentiation and extracellular matrix (ECM) synthesis by promoting Smad2 binding to the ACTA2 promoter [42]. Conversely, *Sirt1* expression is downregulated in AF patients, and this reduction is associated with exaggerated fibrosis. Functional activation of *Sirt1* suppresses the TGF-β1/Smad pathway in atrial fibroblasts, thereby alleviating fibrotic responses [43]. The epigenetic reader *BRD4* is also markedly elevated in AF, stimulating fibroblast proliferation, ECM production and endothelial-mesenchymal transition (EndMT) via transcription factors including Snail and Twist [44,45]. Furthermore, studies in related cardiac pathologies have shown that HDAC-mediated modifications facilitate fibrosis by facilitating the recruitment of *BRD4* to the regulatory regions of fibrosis-related genes, thereby enhancing ECM protein expression and impairing tissue compliance [52]. In chronic hypertension, myocardial activation of HDAC6 has been specifically shown to aggravate atrial fibrosis and interstitial remodeling, thereby directly contributing to the arrhythmogenic structural substrate of AF [46]. Additionally, histone modifications contribute to cardiomyocyte hypertrophy, as illustrated by Ang-II-induced phosphorylation and nuclear export of HDAC4 and HDAC5*.* Such events abolish the inhibition of hypertrophy-related genes and elevate activating histone acetylation marks (H4ac, H3K27ac), thereby facilitating proarrhythmic structural alterations [47].

### 4.2 Histone Modifications Associated With AF Electrical Remodeling

Histone modifications mediate atrial electrical remodeling in AF by regulating ion channel expression and calcium homeostasis, with HDACs serving as core regulators. In AF, HDAC2 downregulation modulates potassium channel transcription both through direct binding to gene regulatory regions and via antagonistic interaction with the repressor NRSF, leading to extended action potential duration [48,49]. The atrial-specific potassium channel KCNN1 also undergoes chamber-specific remodeling that is distinctly regulated by HDACs [53].

Cardiomyocyte calcium handling is also modulated by histone modifications. Hyperactivation of Class I HDACs (e.g., HDAC3) compromises Ca^2+^ handling and decreases Ca^2+^ transient amplitude, while Class IIa HDACs (e.g., HDAC5) counteract this effect by modulating MEF2 activity. Nuclear localization of HDAC5 alleviates pacing-induced Ca^2+^ handling dysfunction and action potential abnormalities [54]. Additionally, chronic HDAC6 activation disrupts electrical conduction by causing heterogeneous connexin (CX40, CX43) distribution and slowing conduction velocity, directly increasing AF susceptibility [46].

### 4.3 Potential Biomarkers and Therapeutic Targets for Histone Modifications in AF

Molecules involved in histone modification serve as promising candidates for both diagnostic biomarkers and therapeutic targets in AF. Therapeutically, *EZH2* inhibition alleviates Ang-II-induced atrial fibrosis and reduces AF susceptibility [42], while* BRD4* inhibition reduces fibroblast activation and ECM overproduction [44,45]. Conversely, Sirt1 activation inhibits fibrogenesis through the TGF-β1/Smad pathway [43]. Among the HDAC family, subtype-selective inhibitors exert distinct cardioprotective effects. HDAC3 inhibition contributes to maintaining calcium homeostasis and contractility, whereas HDAC6 inhibition ameliorates electrical remodeling and Ca^2+^ handling defects [54,55,56]. Clinically applied HDAC inhibitors such as valproic acid also mitigate atrial remodeling and lower AF inducibility in animal models [57]. However, some HDAC inhibitors may have proarrhythmic effects, thus their clinical translation requires a rigorous benefit-risk assessment [58]. Collectively, these findings underscore the potential of histone modification-targeted strategies for precision therapy in AF.

## 5. Non-Coding RNA and AF

**Fig. 3.**
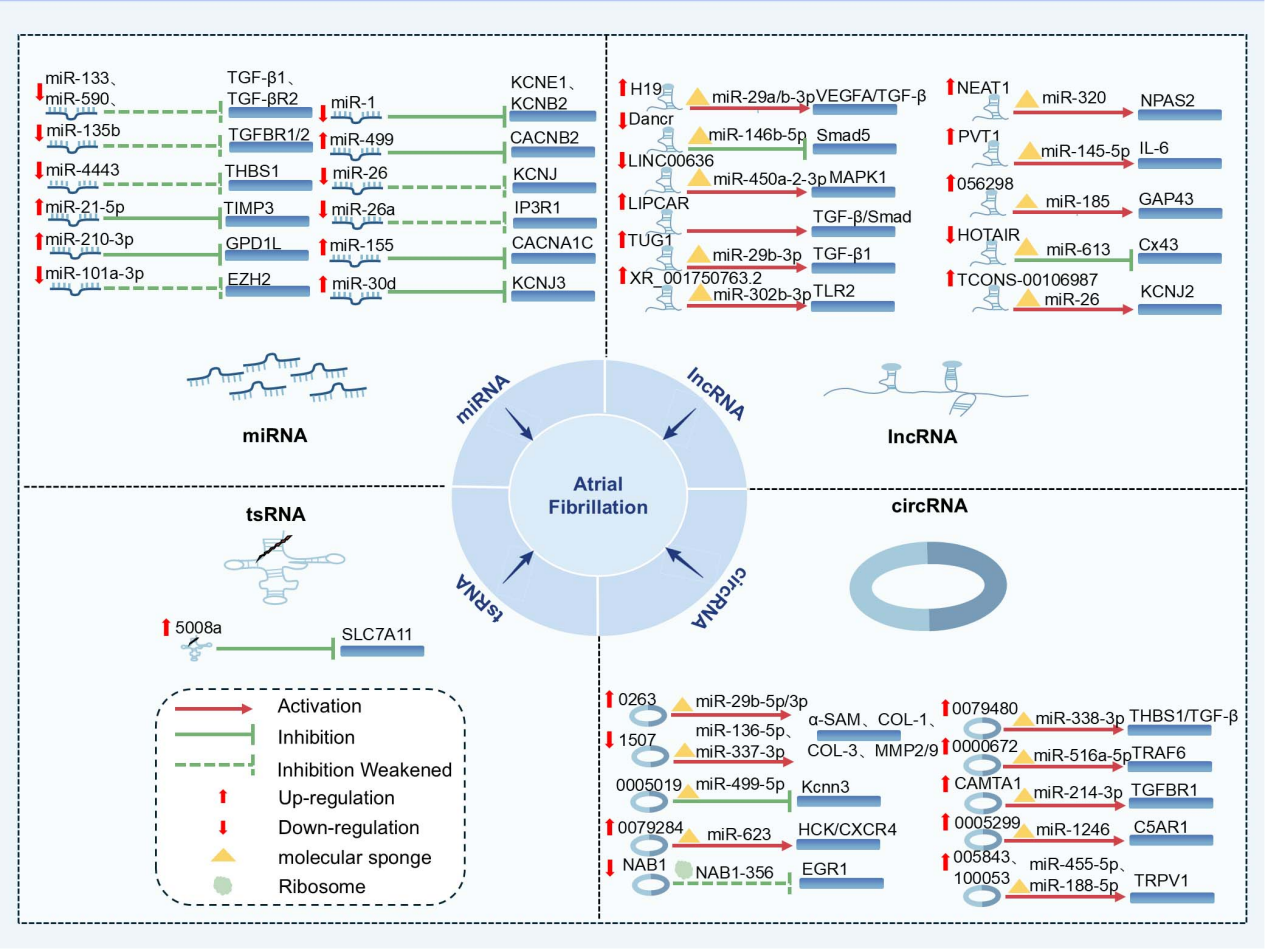
**A network of molecular mechanisms of non-coding RNA-mediated epigenetic regulation in the development of AF. **Abbreviations are defined as follows: α-SMA, Alpha-smooth muscle actin; C5AR1, Complement C5a receptor 1; *CACNA1C*, Calcium voltage-gated channel subunit alpha1 C; *CACNB2*, Calcium voltage-gated channel auxiliary subunit beta 2; CAMTA1, Calmodulin binding transcription activator 1; COL-1, Collagen type I; COL-3, Collagen type III; CX43 (GJA1), Gap junction protein alpha 1 (Connexin 43); EGR1, Early growth response protein 1; *EZH2*, Enhancer of zeste 2 polycomb repressive complex 2 subunit; GAP43, Growth associated protein 43; GPD1L, Glycerol-3-phosphate dehydrogenase 1-like; HCK, Hematopoietic cell kinase; IL-6, Interleukin 6; IP3R1 (ITPR1), Inositol 1,4,5-trisphosphate receptor type 1; *KCNB2*, Potassium voltage-gated channel subfamily B member 2; *KCNE1*, Potassium voltage-gated channel subfamily E regulatory subunit 1; KCNJ, Potassium inwardly rectifying channel subfamily J;* KCNJ2*, Potassium inwardly rectifying channel subfamily J member 2; *KCNJ3*, Potassium inwardly rectifying channel subfamily J member 3; *KCNN3*, Potassium calcium-activated channel subfamily N member 3; MAPK1, Mitogen-activated protein kinase 1; MMP2, Matrix metalloproteinase 2; MMP9, Matrix metalloproteinase 9; NAB1, NGFI-A binding protein 1; NPAS2, Neuronal PAS domain protein 2; SLC7A11, Solute carrier family 7 member 11; Smad5, Mothers against decapentaplegic homolog 5; TGF-β1, Transforming growth factor beta 1; TGF-βR2 (TGFBR2), Transforming growth factor beta receptor 2; TGFBR1, Transforming growth factor beta receptor 1; THBS1, Thrombospondin 1; TIMP3, TIMP metallopeptidase inhibitor 3; TLR2, Toll-like receptor 2; TRPV1, Transient receptor potential vanilloid 1; VEGFA, Vascular endothelial growth factor A.

Non-coding RNAs (ncRNAs) are RNA molecules that do not encode proteins. They play critical roles in cellular homeostasis, development, and disease pathogenesis by regulating processes such as gene transcription, translation, and RNA processing [59]. Based on transcript length, ncRNAs are broadly categorized into short ncRNAs (e.g., miRNAs, tsRNAs) and long ncRNAs (lncRNAs), with circular RNAs (circRNAs) constituting a functionally stable subclass due to their covalently closed structure [59,60]. In AF, these ncRNAs contribute to atrial remodeling through distinct mechanisms: miRNAs directly target and repress mRNA expression [61] (Table 3, Ref. [9,10,62,63,64,65,66,67,68,69,70,71,72,73,74,75,76,77,78,79,80,81,82,83,84,85,86,87,88]); lncRNAs and circRNAs often act as competing endogenous RNAs (ceRNAs), sequestering miRNAs via molecular sponge activity to form complex regulatory networks [61] (Table 4, Ref. [89,90,91,92,93,94,95,96,97,98,99,100,101,102,103,104,105,106,107]); and the recently identified tsRNAs, derived from tRNA cleavage, participate in AF progression through post-transcriptional regulation [108,109] (Table 5, Ref. [109,110,111,112]). Collectively, they constitute a complex, multi-layered regulatory network that drives AF progression through the targeting of ion channels, fibrosis-associated factors, and inflammatory mediators, thereby disrupting electrical conduction, promoting fibroblast proliferation, and sustaining a pro-inflammatory milieu (Fig. 3).

**Table 3.** **Regulatory roles of miRNAs in epigenetics of AF**.

miRNA	Expression change in AF	Study model	Target gene/Pathway	Regulated pathological process (Promotes)	References
miR-133, miR-590	↓	Nicotine-induced canine AF model and smoking AF patients	TGF-β1, TGF-βRII	Structural Remodeling	[62]
miR30-c	↓	TGF-β1-induced fibrotic cell model, rat ACC model	TGF-βRII	Structural Remodeling	[63]
miR-21	↑	Rapid atrial pacing (RAP) rabbit model	Smad7	Structural Remodeling	[64]
miR-29b	↓	AF rat model	TGFβRΙ	Structural Remodeling	[65]
miR-135b	↓	Atrial tissue from AF patients	TGFBR1, TGFBR2	Structural Remodeling	[66]
miR-23b-3p, miR-27b-3p	↑	Human atrial fibroblasts, atrial appendage tissue from AF patients	TGFBR3	Structural Remodeling	[67]
miR-4443	↓	Plasma from AF patients, human ventricular fibroblasts THBS1	THBS1	Structural Remodeling	[68]
miR-1202	↑	TGFβ-1-induced human cardiac fibroblasts	nNOS	Structural Remodeling	[71]
miR-135a	↓	Plasma from AF patients, ACh-CaCl_2_-induced AF rat model	Smad3	Structural Remodeling	[72]
miR-21-5p	↑	Rapid atrial pacing model	TIMP3	Structural Remodeling	[73]
miR-210-3p	↑	AF patients, exosomes from rapidly paced cardiomyocytes	GPD1L	Structural Remodeling	[69]
miR-124-3p	↑	Plasma exosomes from AF patients	AXIN1	Structural Remodeling	[70]
miR-101a-3p	↓	Atrial tissue from AF patients	*EZH2*	Structural/Electrical Remodeling	[74]
miR-425-5p	↓	Plasma and atrial tissue from AF patients, ACh-CaCl_2_-induced AF mouse model	CREB1	Structural Remodeling	[75]
miR-205-5p	↓	AF patients, high-fat diet-induced mouse atrial fibrosis model	EHMT2/IGFBP3 axis	Structural Remodeling	[76]
miR-223-3p	↑	AF patients, the isoproterenol-induced AF model in aged rats	FOXO3	Structural Remodeling	[77]
miR-23a-3p	↑	Rapid pacing canine model, rapidly paced H9C2 cells	SLC7A11	Structural Remodeling	[78]
miR-155	↑	Atrial myocytes from AF patients, transgenic mice	*CACNA1C*	Electrical Remodeling	[10]
miR-21, miR-208b	↑	Chronic AF patients, HL-1 cells	*CACNA1C*, *CACNB2*	Electrical Remodeling	[79,81]
miR-328	↑	Canine and human AF models	*CACNA1C*, *CACNB1*	Electrical Remodeling	[9]
miR-499	↑	Atrial tissue from permanent AF patients	*CACNB2*, *KCNN3*(SK3)	Electrical Remodeling	[80]
miR-1, miR-26a/b	↓	Human left and right atrial tissue slices	IK1 channel	Electrical Remodeling	[82]
miR-199a-5p, miR-22-5p	↑	Patients with HFrEF and AF	L-type Ca^2+^ channel, NCX、Connexin-40	Electrical Remodeling	[83]
miR-26a	↓	Rapid pacing-induced canine AF model	IP3R1	Electrical Remodeling	[85]
miR-135	↓	Mouse AF model	NLRP3 inflammasome	Structural/Electrical Remodeling	[86]
miR-26	↓	Atrial tissue from animal AF models and AF patients	KCNJ (KIR2.1)	Electrical Remodeling	[87]
miR-1	↑	Rapid right atrial pacing rabbit model	*KCNE1*, *KCNB2*	Electrical Remodeling	[84]
miR-30d	↑	Cardiomyocytes from persistent AF patients	*KCNJ3* (Kir3.1)	Electrical Remodeling	[88]

Abbreviations are defined as follows: ACC, Acetylcholine Chloride; ACh-CaCl_2_, Acetylcholine and Calcium Chloride; AF, Atrial Fibrillation; AXIN1, Axin 1;* CACNA1C*, Calcium Voltage-Gated Channel Subunit Alpha1 C; CACNB1, Calcium Voltage-Gated Channel Auxiliary Subunit Beta 1; *CACNB2*, Calcium Voltage-Gated Channel Auxiliary Subunit Beta 2; CREB1, CAMP Responsive Element Binding Protein 1; EHMT2, Euchromatic Histone Lysine Methyltransferase 2; *EZH2*, Enhancer of Zeste Homolog 2; FOXO3, Forkhead Box O3; GPD1L, Glycerol-3-Phosphate Dehydrogenase 1 Like; HFrEF, Heart Failure with Reduced Ejection Fraction; IGFBP3, Insulin Like Growth Factor Binding Protein 3; IK1, Inward Rectifier Potassium Current; IP3R1, Inositol 1,4,5-Trisphosphate Receptor Type 1; *KCNB2*, Potassium Voltage-Gated Channel Subfamily B Member 2; *KCNE1*, Potassium Voltage-Gated Channel Subfamily E Regulatory Subunit 1; KCNJ, Potassium Inwardly Rectifying Channel Subfamily J; *KCNN3*, Potassium Calcium-Activated Channel Subfamily N Member 3; miRNA, MicroRNA; NCX, Sodium-Calcium Exchanger; nNOS, Neuronal Nitric Oxide Synthase; NLRP3, NOD-, LRR- and Pyrin Domain-Containing Protein 3; RAP, Rapid Atrial Pacing; SLC7A11, Solute Carrier Family 7 Member 11; Smad, Mothers Against Decapentaplegic Homolog; TGF-β1, Transforming Growth Factor Beta 1; TGFBR, Transforming Growth Factor Beta Receptor; THBS1, Thrombospondin 1; TIMP3, TIMP Metallopeptidase Inhibitor 3.

**Table 4.** **Non-coding RNAs regulating AF structural and electrical remodeling via the ceRNA mechanism**.

sncRNA		Changes in sncRNA expression in AF	Sponged miRNA (ceRNA Axis)	Target gene/Pathways regulated	Net effect on pathology (Promotes)	References
LncRNA						
	LncRNA H19	↑	miR-29a-3p, miR-29b-3p	VEGFA/TGF-β axis	Structural Remodeling	[89]
	LncRNA Dancr	↓	miR-146b-5p	Smad5	Structural Remodeling	[90]
	LncRNA LIPCAR	↑	Directly regulates	TGF-β/Smad	Structural Remodeling	[91]
	LncRNA TUG1	↑	miR-29b-3p	TGF-β1	Structural Remodeling	[92]
	LncRNA XR_001750763.2	↑	miR-302b-3p	TLR2 inflammatory pathway	Structural Remodeling	[93]
	LINC00636	↓	miR-450a-2-3p	MAPK1	Structural Remodeling	[94]
	LncRNA NEAT1	↑	miR-320	NPAS2	Structural Remodeling	[95]
	LncRNA PVT1	↑	miR-145-5p	IL-6	Structural Remodeling	[96]
	LncRNA TCONS-00106987	↑	miR-26	*KCNJ2*	Electrical Remodeling	[97]
	LncRNA HOTAIR	↓	miR-185	Cx43	Electrical Remodeling	[98]
	LncRNA 056298	↑	miR-185	GAP43	Electrical Remodeling	[99]
circRNA						
	circ_0079480	↑	miR-338-3p	THBS1/TGF-β1/Smad3	Structural Remodeling	[100]
	hsa_circ_0000672	↑	miR-516a-5p	TRAF6	Structural Remodeling	[101]
	circCAMTA1	↑	miR-214-3p	TGFBR1	Structural Remodeling	[102]
	circ_0005299	↑	miR-1246	C5AR1	Structural Remodeling	[105]
	circ_0079284	↑	miR-623	HCK/CXCR4	Structural Remodeling	[105]
	circRNA_0263	↑	miR-29b-5p, miR-496-5p	α-SMA, COL-1, COL-3, MMP2/9	Structural Remodeling	[106]
	circRNA_1507	↓	miR-136-5p, miR-337-3p	α-SMA, COL-1, COL-3, MMP2/9	Structural Remodeling	[106]
	circNAB1	↓	Encodes protein NAB1-356	EGR1	Structural Remodeling	[107]
	mmu_circ_0005019	Not specified	miR-499-5p	*Kcnn3*	Structural/Electrical Remodeling(inhibition)	[103]
	hsa_circ_100053, hsa_circ_005843	↑	miR-455-5p, miR-188-5p	TRPV1, SPON1	Structural/Electrical Remodeling	[104]

↑: upregulation; ↓: downregulation. Abbreviations are defined as follows: AF, Atrial Fibrillation; α-SMA, α-Smooth Muscle Actin; C5AR1, Complement C5a Receptor 1; COL, Collagen; CXCR4, C-X-C Motif Chemokine Receptor 4; Cx43, Connexin 43; ceRNA, Competing Endogenous RNA; circRNA, Circular RNA; EGR1, Early Growth Response 1; GAP43, Growth Associated Protein 43; HCK, HCK Proto-Oncogene, Src Family Tyrosine Kinase; IL-6, Interleukin-6; KCNJ2, Potassium Inwardly Rectifying Channel Subfamily J Member 2; Kcnn3, Potassium Calcium-Activated Channel Subfamily N Member 3; LncRNA, Long Non-Coding RNA; MAPK1, Mitogen-Activated Protein Kinase 1; miRNA, MicroRNA; MMP, Matrix Metallopeptidase; NAB1, NGFI-A Binding Protein 1; NPAS2, Neuronal PAS Domain Protein 2; sncRNA, Small Non-Coding RNA; Smad, SMAD Family Member; SPON1, Spondin 1; TGF-β, Transforming Growth Factor Beta; TGFBR1, Transforming Growth Factor Beta Receptor 1; THBS1, Thrombospondin 1; TLR2, Toll Like Receptor 2; TRAF6, TNF Receptor Associated Factor 6; TRPV1, Transient Receptor Potential Cation Channel Subfamily V Member 1; VEGFA, Vascular Endothelial Growth Factor A.

**Table 5.** **tsRNAs associated with AF and their functional roles**.

tsRNAs	Study model	Expression change in AF	Target gene/Pathways regulated	Net effect on oathology (Promotes)	References
tsRNA-5008a	Mouse AF model	↑	SLC7A11/Ferroptosis pathway	Structural Remodeling	[110]
mt_tRNA-Val-TAC_CCA_end	Aging mouse atria	↑	Oxidative stress, inflammation, Smad3/Mkl1/Ndufs6	Structural Remodeling	[111]
tsRNA-5006c-Lys-CTT	Human plasma	↑	Unidentified	Prognostic association	[109]
191 differentially expressed tsRNAs	Human atrial appendage	Dysregulated	Calcium signaling, AMPK, insulin pathways (bioinformatic prediction)	Not functionally validated	[109]
272 differentially expressed tsRNAs	Human epicardial adipose tissue	146↑	Glycosaminoglycan biosynthesis, AMPK, insulin signaling (bioinformatic prediction)	Not functionally validated	[112]

↑: upregulation. Abbreviations are defined as follows: AF, atrial fibrillation; AMPK, AMP-activated protein kinase; SLC7A11, solute carrier family 7 member 11; tsRNAs, transfer RNA-derived small RNAs.

### 5.1 MicroRNAs and AF

MicroRNAs (miRNAs) is a class of endogenous ncRNAs approximately 22 nucleotides in length that regulate gene expression by binding to the 3′ untranslated region (3′UTR) of target mRNAs [113]. In AF, miRNAs exhibit distinct expression patterns: RNA-seq of atrial tissues from patients with persistent AF identified 20 differentially expressed miRNAs (9 upregulated, 11 downregulated) [114], while 19 miRNAs were markedly upregulated in large extracellular vesicles from non-valvular AF patients [115]. These findings support the region-specific and AF subtype-specific dysregulation of miRNAs, suggesting their critical involvement in AF pathophysiology.

#### 5.1.1 MiRNAs Associated With AF Structural Remodeling

miRNAs regulate atrial fibrosis chiefly through the TGF-β/Smad signaling pathway. Growing evidence indicates that numerous miRNAs modulate atrial fibrotic progression by targeting core components of this pathway. For instance, the downregulation of miR-133 and miR-590 drives profibrotic TGF-β signaling in both nicotine-induced canine AF models and smoking AF patients [62]. miR-30c suppresses cardiac fibroblast (CF) activation via targeting TGFβRII [63], while miR-21 potentiates TGF-β1/Smad signaling through the repression of Smad7 [64]. MiR-29b and miR-135b target TGFβRΙ and TGFBR1/2, respectively, to attenuate fibrosis [65,66]. Increased expression of miR-23b-3p and miR-27b-3p stimulates Smad3 signaling by targeting TGFBR3 [67], and decreased miR-4443 suppresses the TGF-β1 pathway through the targeting of THBS1 [68]. Additionally, miR-210-3p modulates fibrotic responses through the phosphatidylinositol 3-kinase (PI3K)/Akt pathway [69], miR-124-3p functions via the WNT/β-catenin signaling pathway [70], and miR-101a-3p exerts its regulatory effects by inhibiting *EZH2* [116]. In AF models, upregulated miR-223-3p and miR-23a-3p accelerate fibrotic progression by targeting FOXO3 and triggering ferroptosis, respectively [77,78].

#### 5.1.2 MiRNAs Associated With AF Electrical Remodeling

miRNAs drive AF atrial electrical remodeling via post-transcriptional modulation of ion channel expression and function. Among Ca^2+^ channel-related miRNAs, key regulators including miR-155 [10], miR-21 [79], miR-328 [9] and miR-499 [80], target L-type calcium channel (LTCC) subunits such as *CACNA1C* and *CACNB2*, thereby decreasing the L-type calcium current (ICa-L) amplitude. In chronic AF, upregulation of miR-208b impairs sarcoplasmic/endoplasmic reticulum Ca^2+^-ATPase 2 (SERCA2) activity [81]. During AF, the downregulation of miR-26a induces nuclear Ca^2+^ overload through the targeting of Inositol 1,4,5-Trisphosphate Receptor Type 1 (IP3R1) [85], whereas the ectopic overexpression of miR-135 inhibits intracellular Ca^2+^ release and NLRP3 inflammasome activation, thereby reducing AF vulnerability [86].

Potassium channel regulation is also critical. The downregulation of miR-26 increases *KCNJ2* expression and IK1 density, thereby facilitating AF initiation [87]. A separate study using a rapid right atrial pacing rabbit model demonstrated that the upregulation of miR-1 represses *KCNE1* and *KCNB2* expression, enhances the slow delayed rectifier potassium current (IKs), abbreviates the atrial effective refractory period (AERP), and augments AF inducibility [117]. In permanent AF, miR-499 downregulates SK3 expression by targeting *KCNN3* [80], while in persistent AF, miR-30d diminishes *I*_K,ACh_ via the inhibition of *KCNJ3* in a Ca^2+^/PKC-dependent manner, uncovering an alternative regulatory mechanism [88].

#### 5.1.3 Potential Biomarkers and Therapeutic Targets for MiRNAs in AF

MiRNAs hold great promise as both diagnostic biomarkers and therapeutic targets for AF, with significant potential in diagnosis, prognosis and recurrence prediction. A meta-analysis identified multiple miRNAs including miR-4798, miR-133a and miR-150 as robustly associated with AF (pooled OR = 2.51, 95% CI: 1.99–3.16) [118]. In addition to diagnosis, increased levels of miRNAs including miR-21, miR-27b and miR-206 are strongly correlated with AF recurrence following catheter ablation [119,120], and plasma miR-155 has also been identified as a predictor of AF recurrence after cardioversion, with significantly elevated levels observed in patients who experienced recurrence [121].Further candidates such as miR-208b are elevated in both paroxysmal and persistent AF patients, supporting its potential utility as a serum biomarker [122], whereas exosomal miR-122-5p is significantly upregulated in postoperative AF, highlighting the clinical relevance of exosomal miRNAs for clinical assessment [123]. From a therapeutic perspective, miRNAs such as miR-210-3p, miR-328 and miR-155 serve as attractive therapeutic targets owing to their critical roles in AF-related structural and electrical remodeling. The targeting of these miRNAs may offer a strategy to modulate the underlying arrhythmogenic substrate [9,10,69].

### 5.2 LncRNA and AF

Long non-coding RNAs (lncRNAs) are transcripts over 200 nucleotides in length that lack protein-coding capacity, regulate epigenetic, transcriptional and post-transcriptional processes [124]. In AF, lncRNAs frequently function as competitive endogenous RNAs (ceRNAs) to modulate miRNA activity. Widespread dysregulation of lncRNAs has been consistently documented in independent studies: analysis of peripheral blood mononuclear cells identified 912 differentially expressed lncRNAs and a ceRNA network composed of 108 lncRNAs [125]; examination of atrial tissue detected 82 dysregulated lncRNAs and a 44-lncRNA ceRNA network [93]; and bioinformatics analysis of GEO datasets identified 18 differentially expressed lncRNAs [126]. Collectively, these findings underscore the critical importance of complex ceRNA networks mediated by lncRNAs in AF pathophysiology.

#### 5.2.1 LncRNAs Associated With AF Structural Remodeling

LncRNAs serve as important epigenetic regulators of atrial structural remodeling, primarily through ceRNA mechanisms that modulate fibrotic pathways [125]. Multiple lncRNAs converge on the TGF-β/Smad signaling axis: H19 promotes fibrosis by sponging miR-29a-3p/miR-29b-3p to enhance VEGFA/TGF-β expression [89], lncRNA Dancr exerts anti-fibrotic effects in AF models by binding miR-146b-5p and upregulating Smad5 expression [90]. Similarly, LIPCAR directly enhances TGF-β/Smad signaling [91], and TUG1 upregulates TGF-β1 by sponging miR-29b-3p [92]. Beyond TGF-β, lncRNAs modulate structural remodeling through inflammatory and MAPK pathways: XR_001750763.2 amplifies TLR2-driven inflammation by competing for miR-302b-3p [93], , and LINC00636 suppresses MAPK1-dependent fibrosis through miR-450a-2-3p [94]. Additionally, NEAT1 sponges miR-320 to upregulate NPAS2 [95], and exosomal PVT1 promotes macrophage polarization and ECM remodeling via the miR-145-5p/IL-16 axis [96]. These findings collectively demonstrate that lncRNAs intricately regulate multiple signaling pathways through ceRNA networks to orchestrate the progression of atrial fibrosis.

#### 5.2.2 LncRNAs Associated With AF Electrical Remodeling

LncRNAs are key regulators of AF electrical remodeling through ceRNA mechanisms, influencing ion channel function, connexin expression, and autonomic neural activity. Regarding ion channel regulation, lncRNA TCONS-00106987 is upregulated in AF models, where it sponges miR-26 to increase *KCNJ2* expression, thereby enhancing *I*_K1_ and shortening the AERP [97]. Conversely, lncRNA HOTAIR is downregulated in the atrial tissue of patients with chronic AF; it competitively binds to miR-613 to upregulate Cx43, contributing to impaired electrical coupling [98]. Beyond direct electrical effects, lncRNA 056298 promotes intrinsic cardiac autonomic nerve remodeling and AF susceptibility through the miR-185/GAP43 axis [99].

#### 5.2.3 Potential Biomarkers and Therapeutic Targets for LncRNA in AF

LncRNAs hold dual promise as clinical biomarkers and therapeutic targets in AF. As biomarkers, aberrant expression of AC009509.2 and LINC01781 predicts AF onset [127], while serum exosomal LOC107986997 serves as a diagnostic marker [128]. Additionally, elevated plasma H19 levels are associated with stroke risk in AF patients and improve the predictive power of the CHA_2_DS_2_-VASc score [129]. Conversely, decreased circulating GAS5 levels are useful for early AF diagnosis and predicting recurrence after radiofrequency ablation [130]. Therapeutically, targeting specific lncRNAs represents a promising strategy for AF intervention. Suppressing profibrotic lncRNAs like NEAT1 and TUG1 may attenuate structural remodeling [92,95], while enhancing protective LINC00636 activity could counter fibrosis [94]. Modulation of PVT1 represents another potential approach for regulating ECM remodeling [96]. These findings collectively provide a rationale for developing precision therapies targeting specific ceRNA networks in AF.

### 5.3 circRNA and AF

Circular RNAs (circRNAs) are a class of covalently closed circular ncRNAs generated by back-splicing. Their lack of a 5′ cap and 3′ polyadenylated tail structure confers high stability [131]. In recent years, studies have revealed widespread differential expression of circRNAs in both atrial tissues and peripheral blood of AF patients: comparative analysis of paroxysmal versus persistent AF identified 83 and 99 differentially expressed circRNAs, respectively [132]; right atrial tissues revealed 600 AF-associated circRNAs forming a 30-core ceRNA network [133]; and peripheral blood from postoperative AF patients exhibited 12,834 differentially expressed circRNAs [134]. These results consistently indicate that circRNAs are widely and differentially expressed across AF subtypes, lesion sites, and disease stages, suggesting a key regulatory role in the structural and electrical remodeling of AF.

#### 5.3.1 CircRNAs Associated With AF Structural Remodeling

CircRNAs mainly regulate atrial fibrosis via the competing endogenous RNA (ceRNA) mechanism: circ_0079480 upregulates THBS1 by sponging miR-338-3p, thereby activating the THBS1/TGF-β1/Smad3 pathway and promoting fibroblast activation [100]; hsa_circ_0000672 upregulates TRAF6 by sponging miR-516a-5p, stimulating the TGF-β/MAPK pathway and contributing to left atrial fibrosis [101]; circCAMTA1 relieves the suppression of TGFBR1 by sponging miR-214-3p, and its silencing alleviates atrial fibrosis in mice [102]; the murine homolog mmu_circ_0005019 upregulates *Kcnn3* by sponging miR-499-5p, inhibiting fibroblast proliferation and migration while modulating cardiomyocyte ion channels [103]. Additional circRNAs, including circDGCR8, promote collagen synthesis and fibroblast proliferation [135], while circ_0005299 and circ_0079284 may modulate immune cell infiltration through miR-1246/C5AR1 and miR-623/HCK/CXCR4 axes, respectively [136]. In intermittent hypoxia models, circRNA_0263 and circRNA_1507 influence fibroblast function by competing for miR-29b-5p and miR-496-5p [106].

Notably, specific circRNAs exhibit protein-coding capacity and participate in AF pathogenesis by generating functional proteins through internal open reading frames (ORFs) [137]. For instance, circNAB1 is downregulated in AF, and its translated product, the NAB1-356 protein, binds to the transcription factor EGR1 to govern the expression of Runx1 and Gadd45b, thus decreasing collagen deposition and inflammatory responses and exerting cardioprotective actions [107].

#### 5.3.2 CircRNAs Associated With AF Electrical Remodeling

CircRNAs participate in atrial electrical remodeling through the ceRNA mechanism. For example, overexpression of mmu_circ_0005019 in cardiomyocytes upregulates *Kcnn3* expression by sponging miR-499-5p, thereby modulating potassium ion currents and delaying the progression of AF electrical remodeling [103]. In addition, an integrative analysis based on atrial tissue microarray data from AF patients identified regulatory axes including hsa_circ_100053/miR-455-5p/TRPV1 and hsa_circ_005843/miR-188-5p/SPON1 that may influence AF occurrence by affecting genes related to ion channels or myocardial electrical coupling [104].

#### 5.3.3 Potential Biomarkers and Therapeutic Targets for CircRNAs in AF

CircRNAs hold great promise as both diagnostic biomarkers and therapeutic targets for AF. Specific plasma circRNAs, such as hsa_circ_0070391 and hsa_circ_0003935, demonstrate the ability to discriminate AF patients from healthy controls [138]. The diagnostic potential is further supported by a panel of eight circRNAs (e.g., circRNA_00324, circRNA_17225), which collectively achieve Area Under Curve (AUC) values ranging from 0.68 to 0.93 [139]. In addition to diagnosis, circRNAs possess significant prognostic value. Elevated plasma levels of hsa_circ_0070391 are associated with a lower rate of atrial tachyarrhythmia-free survival after catheter ablation [138]. Similarly, increased circ_81906-RYR2 expression in left auricular tissue serves as an independent risk factor for AF recurrence, and a model based on this marker (AUC = 0.77) effectively stratifies recurrence risk [140]. Furthermore, high plasma hsa_circ_0099734 levels correlate with an increased risk of stroke and all-cause mortality in AF patients [141]. These growing bodies of evidence highlight the emerging role of circRNAs in optimizing risk stratification and facilitating personalized management of AF.

### 5.4 tsRNAs and AF

Transfer RNA-derived small RNAs (tsRNAs), a novel category of short non-coding RNAs generated by site-specific cleavage of precursor or mature tRNAs, are mainly classified into tRNA-derived fragments (tRFs) and tRNA halves (tiRNAs) [142]. They exert regulatory functions by controlling mRNA translation and retrotransposon transcription, thereby participating in diverse physiological and pathological processes [143]. Growing evidence implicates tsRNAs in cardiovascular diseases, including AF. Sequencing analyses have revealed extensive dysregulation of tsRNAs in AF patients, with 191 and 272 tsRNAs abnormally expressed in human atrial and epicardial adipose tissue [109,112]. Bioinformatic enrichment indicates these altered tsRNAs are frequently involved in AF-relevant pathways such as calcium signaling, AMPK activity, and insulin signaling [109,112], directly supporting their functional roles in AF pathogenesis.

Notably, several specific tsRNAs directly participate in AF pathogenesis and are promising therapeutic targets. TsRNA-5008a, which is upregulated in AF, promotes cardiomyocyte ferroptosis and atrial fibrosis by repressing the anti-ferroptosis regulator SLC7A11; its knockdown mitigates these effects and reduces AF susceptibility [110]. Similarly, mitochondrial-derived mt_tRNA-Val-TAC_CCA_end is elevated in aging-related AF models, where it contributes to oxidative stress and inflammation—effects that are reversible with curcumin treatment [111]. Furthermore, tsRNAs such as tsRNA-5006c-Lys-CTT are associated with increased all-cause mortality in AF patients, highlighting their potential as biomarkers [109]. Although research in AF is still developing, these findings support the role of tsRNAs as candidates for elucidating molecular mechanisms and informing future therapeutic and diagnostic strategies.

## 6. Chromatin Remodeling and AF

Chromatin remodeling represents a key form of epigenetic regulation mediated primarily by ATP-dependent chromatin-modifying enzymes, that dynamically regulate chromatin structure and function [144]. Among these, Chromodomain Helicase DNA-Binding (CHD) family proteins constitute critical mediators within this regulatory machinery, encompassing *CHD4*, *CHD6*,* CHD7* and* CHD8*, all of which are ATP-dependent chromatin-modifying enzymes [145,146]. They induce nucleosome sliding, histone eviction or variant incorporation, or even disrupt nucleosome structure in a non-sliding manner (e.g., *CHD6*), significantly altering chromatin accessibility, thereby regulating gene transcription [145,147]. Currently, there are few studies on the association between chromatin remodeling and AF. Existing evidence suggests that this process influences AF pathogenesis by dynamically regulating the transcription of key atrial genes, as demonstrated by the *TBX5-CHD4 *axis, which maintains normal rhythm by promoting a protective gene program, a homeostasis that is disrupted when elevated FOG2 during heart failure inhibits *TBX5*, thereby tipping the balance toward AF [148,149].

Current related research mostly focuses on *CHD4* and related regulatory pathways, while the understanding of the roles of other CHD family members (such as *CHD6*, *CHD7*,* CHD8*, etc.) in AF remains limited. Although they all belong to ATP-dependent chromatin-modifying enzymes, their remodeling mechanisms differ significantly: for example, *CHD6* can disrupt nucleosomes in a non-sliding manner, while *CHD7* and *CHD8* mainly mediate nucleosome sliding and have different requirements for DNA length [145]. These functional differences suggest that they may play distinct roles in atrial myocyte gene regulation and the occurrence of AF [146]. In addition, whether chromatin remodeling and other epigenetic regulatory mechanisms (such as histone modification and DNA methylation) have synergistic effects in AF still requires further investigation [144]. Future studies should combine multi-omics technologies to elucidate the specific mechanisms and regulatory networks of different chromatin remodeling factors (such as other members of the CHD family) in AF, to provide new perspectives for the mechanistic analysis and targeted therapy of AF.

## 7. RNA Methylation and AF

RNA methylation has emerged as a core post-transcriptional modification mechanism in epigenetics. Its regulation depends on three core factors: RNA methyltransferases (“writers”), demethylases (“erasers”), and methylation reader proteins (“readers”) [150,151]. Among these, N^6^-methyladenosine (m^6^A) represents the most prevalent internal chemical modification in eukaryotic RNA [152]. The dynamic equilibrium of m^6^A is co-regulated by methyltransferases (e.g., METTL3/14/16, WTAP), demethylases (e.g., *FTO*, *ALKBH5*), and reader proteins (e.g., YTHDF family, IGF2BP1/2/3) [150], and it regulates multiple aspects of RNA metabolism, including splicing, transport, stability, and translation [151]. Recent studies have begun to delineate how m^6^A dysregulation drives AF pathogenesis through disruptions in cardiomyocyte calcium homeostasis, pro-fibrotic activation of cardiac fibroblasts (CFs), and alterations of the atrial immune landscape.

Both left atrial appendage tissues from AF patients and experimental AF models show significantly decreased METTL3 protein expression and global m⁶A modification levels [153]. Mechanistically, METTL3 downregulation in cardiomyocytes upregulates protocadherin gamma-A10, enhances SERCA2a activity, and disrupts calcium handling, thereby promoting arrhythmogenesis [153]. Concurrently, METTL3 can modulate targets such as the androgen receptor, lncRNA GAS5, and IGFBP3 in an m^6^A-YTHDF2-dependent manner, influencing glycolysis, mitochondrial fission, and proliferation in CFs, thereby participating in atrial fibrosis [154,155,156]. Bioinformatic analyses further reveal distinct m^6^A modification patterns in AF and identify key m^6^A regulators, suggesting that m^6^A may shape the AF immune microenvironment by regulating immune-related genes such as *NCF2* and *HCST *[157]. These findings provide new insights into molecular subtyping and immunotherapy for AF. Further investigation of m^6^A methylation not only advances our understanding of AF mechanisms but also provides new opportunities for its precision diagnosis and targeted therapy.

## 8. Conclusion and Future Directions

This review systematically illustrates the pivotal roles of epigenetic regulation in AF pathogenesis. Mounting evidence shows that multiple epigenetic mechanisms, including DNA methylation, histone modifications, ncRNAs, chromatin remodeling and RNA methylation, collectively form a complex regulatory network. These mechanisms orchestrate both electrical and structural remodeling in AF by modulating ion channel expression, facilitating fibrotic progression and amplifying inflammatory responses. These insights not only advance our understanding of AF pathogenesis but also reveal potential biomarkers for early diagnosis, risk stratification, and prognostic evaluation. Additionally, they define potential molecular targets for the development of innovative therapeutic strategies. Recent reviews by Karakasis et al. [158] and Vinciguerra et al. [159] have comprehensively summarized the epigenetic landscape of AF. The present review extends these works by providing a comprehensive synthesis of under-investigated regulators, including tsRNAs, circRNA-encoded peptides, and RNA methylation, and places greater emphasis on the translational potential of epigenetic biomarkers and targeted therapeutic strategies.

However, critical limitations persist in current AF-related epigenetic research. First, AF epigenetic research is gradually shifting from characterizing individual regulatory mechanisms to dissecting their complex crosstalk in the initiation and maintenance of this arrhythmia. Foundational concepts such as the ceRNA network exemplify this crosstalk; however, a fundamental question remains unresolved: how do DNA methylation, histone modifications, and chromatin coordinately regulate transcriptional programs, and whether this cooperation differs meaningfully across clinical subtypes such as paroxysmal versus persistent AF. The precise mechanisms through which these epigenetic layers drive disease progression in a subtype-specific manner remain unclear. Second, several emerging epigenetic fields, notably RNA methylation and specific histone modifications, remain poorly characterized. Although preliminary associations have been identified, the specific regulatory functions, upstream inducers and functional outcomes of these modifications in atrial tissue are largely unknown, constituting a major research gap. Finally, the clinical translation of epigenetic findings remains inadequate. The majority of putative biomarkers and therapeutic targets are limited to preclinical evaluation, without rigorous validation via large-scale clinical trials that are indispensable for confirming their diagnostic value and therapeutic effectiveness.

Future research should prioritize the application of multi-omics technologies to delineate the interconnected regulatory networks among different epigenetic layers. Subtype-stratified epigenetic studies are required to establish molecular subtyping systems for AF. Furthermore, large-scale clinical studies are essential to validate the diagnostic performance of epigenetic markers and assess the efficacy of targeted therapeutic strategies. Collectively, these endeavors will accelerate the translation of AF epigenetic research from bench to bedside, thus laying a solid foundation for precision medicine in the prevention and management of AF.
